# Morphology of male and female reproductive tract of the ocelot (*Leopardus pardalis*)

**DOI:** 10.1590/1984-3143-AR2020-0010

**Published:** 2020-07-01

**Authors:** Luciana Cristina Machado, Jéssica Rodrigues Orlandin, Rafael Garcia Karam, Felipe Augusto Rós, Daniele dos Santos Martins, Gerlane de Medeiros Costa, Carlos Eduardo Ambrósio

**Affiliations:** 1 Departamento de Medicina Veterinária, Faculdade de Zootecnia e Engenharia de Alimentos, Universidade de São Paulo, Pirassununga, SP, Brasil; 2 Faculdade de Medicina de Ribeirão Preto, Universidade de São Paulo, Ribeirão Preto, SP, Brasil; 3 Laboratório de Zoologia e Morfologia Animal, Universidade do Estado do Mato Grosso, Alta Floresta, MT, Brasil

**Keywords:** morphology, histology, reproductive system, wild felines

## Abstract

The Ocelot (*Leopardus pardalis*) is the largest species of this genus, despite having broad distribution in the Americas; it is included in the main list of endangered species. Their conservation is widely studied, but there is a lack of studies about their morphology. In order to contribute to the knowledge of its reproductive system, five male and female ocelots were examined macro- and microscopically by histological techniques. Macroscopic analysis of the male reproductive system revealed presence of prostate and bulbourethral gland located caudally to the urinary bladder and a penis with small spicules. Microscopically, the testes were encased by the tunica albuginea and divided it into lobules with 5-10 tubules per lobe. In females, macroscopic analysis demonstrated two ovaries position dorsally in the sublumbar region and caudal to the kidneys. The bicornuate uterus is composed by uterine horns (12 to 14 cm in length), which travels from the ovaries in a caudal direction to form a small uterine body (4 cm in length). The ovary analysis revealed, in longitudinal section, medullary region composed of loose connective tissue, a stroma rich in blood vessels, and an external parenchymal region surrounded by a tunica albuginea. The results of the study confirmed the similarity between ocelot's reproductive system as domestic cat's ones and showing for the first time the complete morphological tool to highlight these organs and tissue in this male and female endangered wild felid specie. The present study open venue for other researchers to consider morphological and preservationist features and aimed to help at long-term conservation of wild felines.

## Introduction

The ocelot, *Leopardus pardalis* (Carnivora: Felidae), is a medium-sized mammal, weighing 8-15 kg, with large legs and a slender body, measuring from 50 cm to 1 m in length, males being larger than females (Oliveira and Cassaro, 2005). Its activity pattern is typically nocturnal-crepuscular (Murray and Gardner, 1997; Di Bitetti et al., 2006). Although a good climber, it is a species with terrestrial habits. Its diet includes large and small mammals (Ludlow and Sunquist, 1987; Moreno et al., 2006; Bianchi and Mendes, 2007). The most frequent prey are rats, armadillos, opossums and mice, but it can also feed on anteaters, bats, deer, hares, iguanas and birds (Emmons, 1987; Ludlow and Sunquist, 1987).

It has a wide distribution from the north of Argentina to the south of Texas (Murray and Gardner, 1997). This species can occur in environments such as flood plains, coniferous forests, fields and wet and dry forests (Emmons and Feer, 1997). In Brazil, it occurs in all states with the probable exception of Rio Grande do Sul, occupying the different biomes of the Pantanal, Caatinga, Cerrado, and especially subtropical and tropical forests (Oliveira and Cassaro, 2005). Due to this wide distribution, during the 1960’s and 1970’s, the ocelot was the cat species most exploited by the fur trade (Nowell and Jackson, 1996).

The ocelot currently is included in the Red Book of Brazilian Fauna Threatened by Extinction, elaborated by the IUCN (International Union for Conservation of Nature), where it is classified as of Least Concern - LC (IUCN 3.1) (Caso et al., 2008). However, wild felines such as *Leopardus pardalis* can be considered important for conservation of the ecosystems they live in due to their abundance and ecological significance (Machado et al., 2016). In Brazil, a Management Plan for the ocelot was adopted by IBAMA in 1994 (Portaria 106 of 12/26/95). This plan is coordinated by the Mata Ciliar Association (ACM - Jundiaí - São Paulo - Brazil), with the support of the Zoological Society of Brazil (SZB) and of IBAMA itself, and its objective is “[…] the structuring of a permanent committee for the preservation of the species, coordination of all relevant activities and establishment of strategies for study, management and protection of the ocelot, seeking resources to program them” (ACM, 1998, p. 44-53). However, this ordinance of IBAMA was revoked in 2004.

Research related to the reproduction biology of wild species, in this case more specifically focusing on morphological and ultrastructural studies, is motivated by the need for a greater understanding of reproductive biology, even of non-threatened species, considering the ecological relevance of the species presented here (Machado et al., 2016). Such studies are of great relevance, as they aim to reproduce in captivity more efficiently, reduce neonatal mortality rates in captivity, as well as the constitution and/or enrichment of animal germplasm banks (Mellen, 1991; Brown et al., 1994; Morato and Barnabé, 1998; Moreira et al., 2001; Kleiman, 2010; Micheletti et al., 2012). Therefore, in view of the countless threats to wildlife, it is essential to have more in-depth knowledge about the reproductive biology of target species, with investments in research and actions aimed at *in situ* and *ex situ* conservation.

There are few studies of the ocelot, mainly on its conservation, but there is still a lack of information and data about its morphology with focus in reproductive tools, since structural organization or biobanking (Comizzoli, 2017). Thus, the main objective of this study was to describe the morphology and histology of the organs that compose the male and female reproductive system of *Leopardus pardalis*. Such information could be useful in the reproductive management of the species as well as in comparative studies of wild cat species.

## Material and methods

This research was approved by COBEA (Brazilian College of Animal Experimentation) and the University Ethics Committee (CEP - FZEA) Nº 3351210715 and SISBIO Nº. 49271-1. All the animals used in this study were killed in road traffic accidents in Alta Floresta, Mato Grosso, Brazil. A total of five animal’s adults, three males and two females, were assigned to the Laboratório de Zoologia e Morfologia Animal of the Universidade do Estado de Mato Grosso (UNEMAT). Due to the ecological role of great relevance and the difficulty in obtaining corpses of this species, which are rare to be found, we consider that the sample (n = 5) of this study becomes quite representative.

To study the male and female reproductive apparatus of the ocelot, the animals were fixed by perfusion processes, injecting 10% aqueous formaldehyde solution through the external jugular vein, and immersion in the same fixative, where the pieces remained submerged for a minimum period of 48 hours. Subsequently, the animals were placed in the supine position for dissection of the perineal region. After an incision in the Alba line, the skin was retracted and the organs exposed for dissection and photographic recording with a digital camera. For microscopy, small tissue fragments were fixed in 8% buffered paraformaldehyde solution, and then submitted to standard histological procedures for embedding in paraffin. Blocks were cut in 5 µm sections and stained with hematoxylin-eosin (HE) (Banks, 1992; Bacha and Bacha, 2003).

## Results

### Male reproductive system of the ocelot

**Figure 1:**
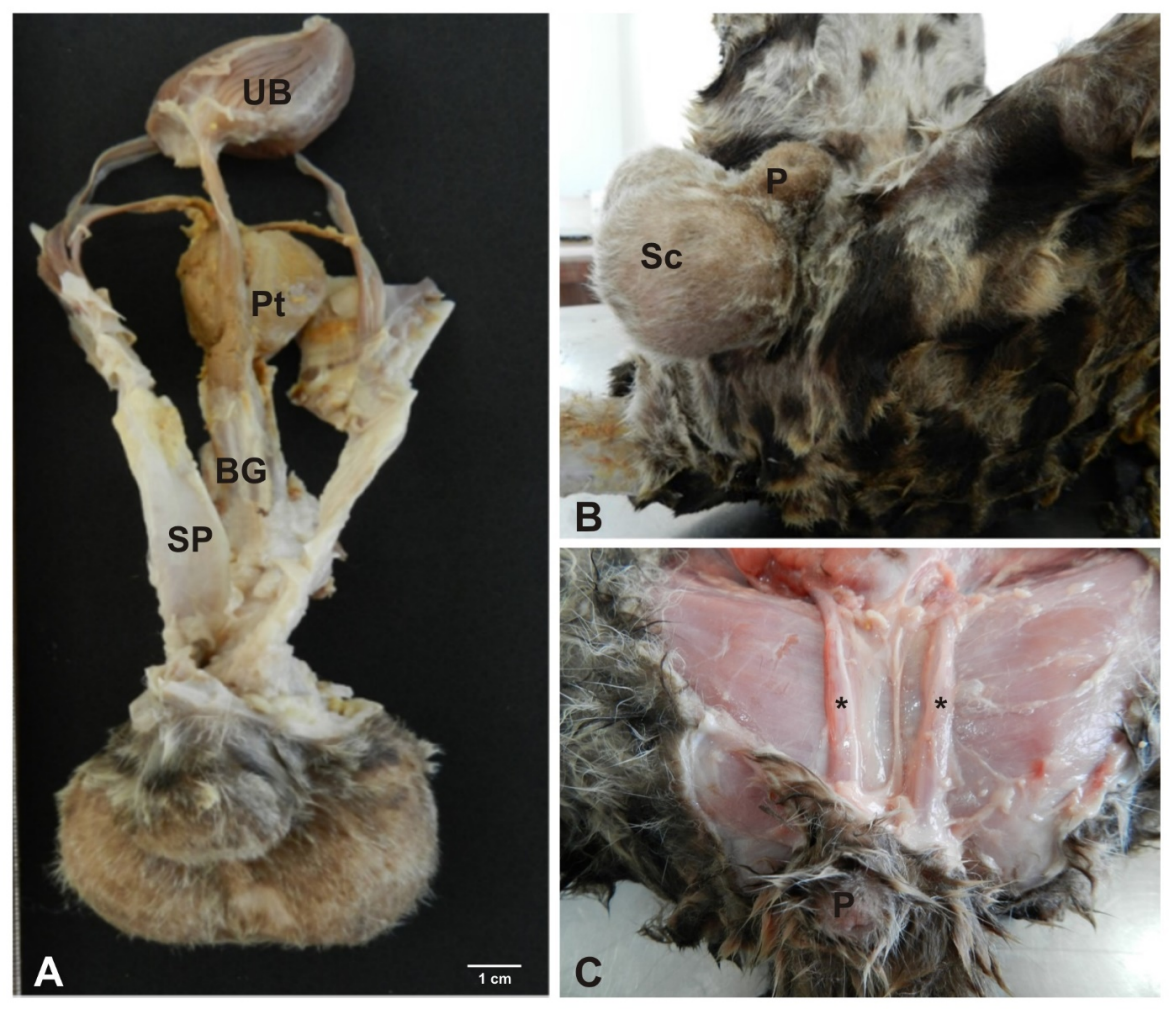
Photograph of the reproductive tract of a male ocelot. (A) the urinary bladder (UB), prostate (Pt), bulbourethral gland (BG), spermatic funiculus (SP) and penis (P); (B) scrotum (Sc) and penis (P); (C) penis (P) and spermatic funiculus (*).

The male reproductive system of *Leopardus pardalis* consists of the following structures: scrotum, penis, testes, vas deferens and epididymis. Also included in this system are the accessory bulbourethral and prostate glands, with absence of vesicular glands (Figures1and2).

**Figure 2:**
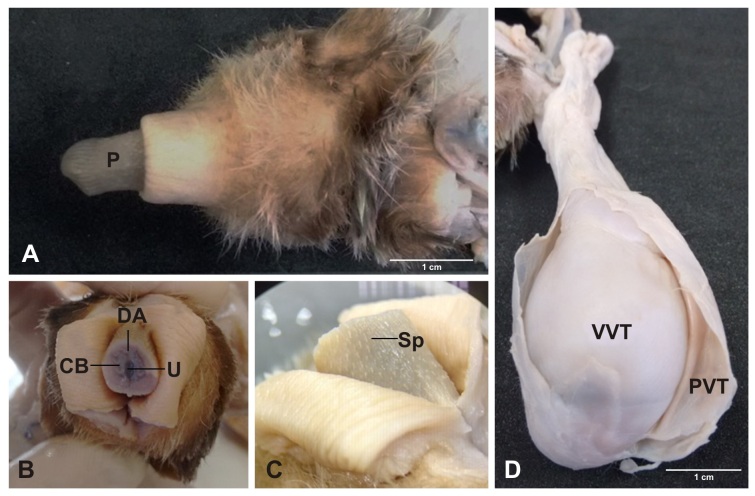
Photograph of the reproductive tract of a male ocelot. (A) penis (P); (B) cavernous body (CB), deep artery (DA) and urethra (U); (C) spicules (Sp); (D) visceral vaginal tunic (VVT) and parietal vaginal tunic (PVT).

### Scrotum

The scrotum with a perineal location was positioned as an extension of the skin of the abdominal region in the pelvic region, as a membranous pocket, divided by a median septum, covered by hairs that harbored the testes, epididymides and the vas deferens (Figure 1B).

### Testis

The paired testes were separated by the scrotal septum. With ovoid shape and rounded contours, they presented a concave border laterally and a straight border positioned medially. They were covered by a fibrous outer membrane of whitish pink color, the visceral vaginal tunic (Figure 2D). Microscopically, the testes were encased by the tunica albuginea, a thick capsule of dense connective tissue, especially at the dorsal surface, which is the mediastinum, through which the fibrous septa that penetrate the testis divided it into lobules. Each lobule was occupied by seminiferous tubules, with 5-10 tubules per lobe (Figures 33 B).

**Figure 3:**
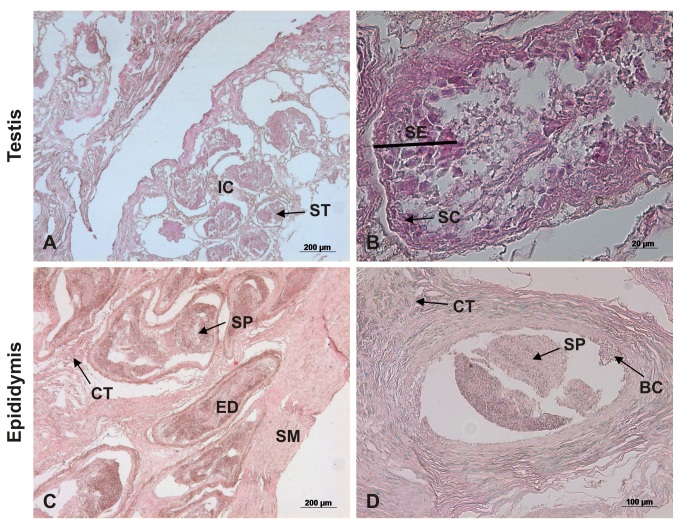
Photomicrograph of the testis and epididymis of an ocelot. (A) note the Interstitial cell (IC); Seminiferous tubules (ST); (B) Seminiferous epithelium (SE); Sertoli cells (SC); (C and D) Connective tissue (CT); Epididymal duct (ED); Smooth muscle (SM); Basal cells (BC); Spermatozoids (SP). Hematoxylin/eosin technique, bar 20 and 200µm.

### Spermatic cord

The spermatic cord consisted of the vas deferens, cremaster muscle, visceral lamina of the vaginal tunica and the pampiniform plexus, composed of testicular veins and arteries. Stretching ventrally through the inguinal ring, it was positioned laterally to the penis, reaching up to the testes (Figures 11B).

### Epididymis

Covered by a thin serous membrane and located on the medial borders of the testes, were the epididymides, comprising head, body and tail. The most prominent portion, the head of the epididymis, was located in the cranial portion of the gonad, firmly attached to the vaginal tunica. The body extended through the ventral region of the testis, reaching the tail at the caudal end of the testis, from which the vas deferens protruded cranially (Figure 2D). Microscopically the epididymal duct and the basal cells with their nuclei were observed. It was also observed that the epididymis consists of basal cells, loose connective tissue, smooth muscle and a cylindrical pseudo-stratified epithelium (Figures 33D).

### Prostate and bulbourethral gland

The prostate was composed of a large, irregularly shaped, compact mass located caudal to the urinary bladder and cranial to the bulbourethral gland. It covered almost the entire pelvic portion of the urethra. The paired bulbourethral glands, with a rounded shape, were located posterior to the pelvic urethra and caudal to the prostate. In the image, the respective structures could not be seen (Figure 1A).

### Foreskin and penis

The foreskin presented as a thick layer of epithelial tissue, covered by hair, with an orifice facing the ventral region, through which the urine and the semen are ejected. The penis was divided into tail, body and glans, and small spicules were observed. In cross section could be seen the cavernous bodies surrounded by tunica albuginea, erectile tissue and deep artery (Figures 11C and 2A).

### Female reproductive system of the ocelot

**Figure 4:**
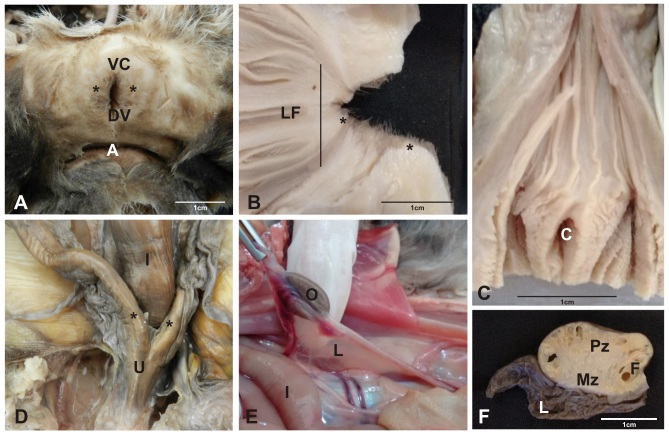
Photograph of female reproductive tract of ocelot. (A) vulvar lips (*), ventral commissure (VC), dorsal commissure (DC) and anus (A); (B) **Figure to** observe longitudinal folds (LF) of the cervix and vagina and vulvar lips (*); (C) External urethral ostio (C); In (D), note intestine (I), uterine horns (*) and uterine body (U); (E) note the ovary (O), broad ligament of the ovary (L) and intestine (I). In (F), cross section of ovary. Note the parenchymal zone (Pz), medullar zone (Mz), tertiary follicle (F) and mesosalpinx (L).

The female reproductive system of *Leopardus pardalis* consists of the following structures: ovary, oviducts, uterus, cervix, vagina, clitoris and vulva (Figure 4).

### Ovary

**Figure 5:**
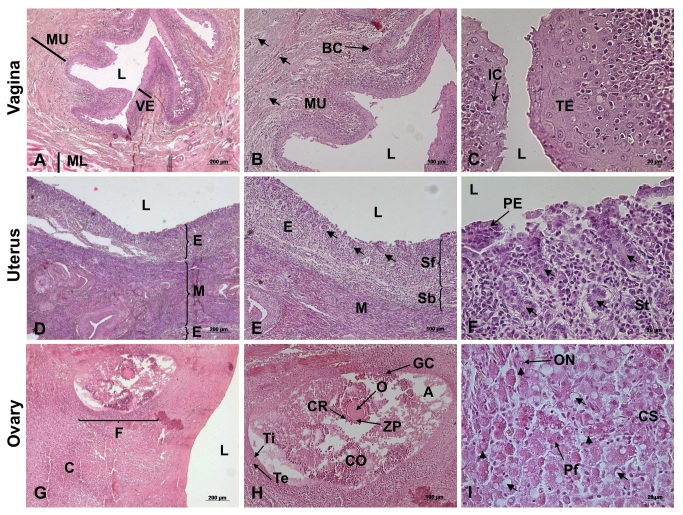
Photomicrograph of female reproductive tract of ocelot. Vagina: (A) Lumen (L), Vaginal epithelium (VE), Muscular layer (ML) and Mucus layer (MU); (B) Lumen (L), Basal cells (BC), Mucus layer (MU) and Blood Vessel (arrow); (C) Lumen (L), Intermediate cells (IC), Transitional Epithelium (TE) and Blood Vessel (arrow). Uterus: (D) Lumen (L), Endometrium (E) and Myometrium (M); (E) Lumen (L), Endometrium (E), Myometrium (M), Stratum basalis (Sb), Stratum functionalis (Sf) and Endometrial gland (arrow); (F) Lumen (L), Pseudostratified epithelium (PE), Endometrial stroma (St) and Endometrial gland (arrow). Ovary: (G) Lumen (L), Cortex (C) and Follicle (F); (H) Oocyte (O), Corona radiata (CR), Zona pellucida (ZP), Antrum (A), Granulosa cells (GC), Cumulus oophofus (CO), Theca interna (Ti), Theca externa (Te) and Primordial follicle (arrow head); (I) Primary follicle (Pf), Cortical stroma (CS), Oocyte nuclei (ON), Atretic follicle (arrow). Hematoxylin/eosin technique, bar 20, 100 and 200µm.

The paired ovaries were positioned dorsally in the sub lumbar region and caudal to the kidneys. They had a curved expanded shape with the concave face downwards, similar to a kidney bean. They were about 1.5 cm in length (Figures 44F). In longitudinal section, there was a medullary region composed of loose connective tissue, a stroma rich in blood vessels, and an external parenchymal region surrounded by a tunica albuginea (Figure 5).

### Oviduct

The paired oviducts were pinkish-white in color and measured 6 and 10 cm. The uterine tube is surrounded by a peritoneal tissue derived from the broad ligament of the uterus, called mesosalpinx, which is subdivided into: the infundibulum of the uterine tube, ampulla and isthmus (Figure 4D). The uterine tube makes an opening in the internal uterine ostium, being irrigated by the arteries: ovarian and uterine (Figures 555I).

### Uterus

The uterus consists of a cervix, a uterine body and two uterine horns (bicornuated). The uterine body is positioned between the ascending colon and the urinary bladder. The uterine body was small, about 4 cm in length (Figure 4D). The horns extended to the ovarian pouch and measured 12 and 14 cm in length. Microscopically the presence of vascular extract, endometrial glands, glandular epithelium and pseudo-stratified myometrium epithelium could be shown (Figures 555F).

### Cervix and vagina

Macroscopically, the cervix presented circular longitudinal folds, in annular shape. Internally, it had a thick wall of smooth muscle. It terminated at the external uterine ostium, communicating with the vagina, where the longitudinal folds of the mucosa formed (Figures4B 4C). The vagina extended to the external urethral ostium a had a thick wall composed of smooth musculature with large lips filled by a dense layer of adipose tissue. Microscopically, the vagina comprised the muscular layer, mucus layer, vaginal epithelium basal cells, transitional epithelium and intermediate cells (Figures 555C).

### Vulva and clitoris

The vulva comprised the vulvar lips, which joined together dorsally and ventrally. The ventral clitoris was the most prominent part of this organ. The dorsal part had more rounded contours (Figures 44C).

## Discussion

### Male reproductive system of the ocelot

A macroscopic description of the male reproductive system of *Leopardus pardalis* was previously given by Carneiro et al. (2010), as the penis, scrotum, testis, epididymis, vas deferens and accessory genital glands such as a bulbourethral gland and prostate are present, but not the vesicular glands. The latter are also absent in domestic cats (Godinho, 1999; Dyce et al., 2010) and in wild species as *Puma yaguaroundii* (Rocha et al., 2017). *Leopardus pardalis* has relatively small testes (Sarti, 2006; Carneiro et al., 2010) with the same anatomy as puma (*Puma concolor*) (Mansfield and Land, 2002; Guião-Leite, 2002), jaguar (*Panthera onca*) (Azevedo, 2004) and *Puma yaguaroundii* (Rocha et al., 2017). The scrotum is positioned in the perineal region, lateral to the penis as described by Carneiro et al. (2010) for ocelot and similar to the domestic cat (Getty, 1987). Microscopically, the albuginea tunica in the examined testes of *Leopardus pardalis* is thick and consists of the presence of dense connective tissue moderately modeled, with considerable amounts of collagen fibers and discrete elastic fibers, with emission of thin septa into the parenchyma testicular. The findings corroborate the research conducted by Silva et al. (2009) on domestic felines. As described by Carneiro et al. (2010), in *Leopardus pardalis* the vas deferens is a long and thin structure, similar to that of the domestic cat (Getty, 1987). As shown by Junqueira and Carneiro (2015), the body of the epididymis extends through the ventral region of the testis, reaching the tail at the caudal end of the testis, from which the vas deferens cranially projects, in the same way as in domestic carnivores. Microscopically, the epididymal duct and the basal cells with their nuclei were demonstrated. It was also observed that the epididymis is composed of smooth muscle and a cylindrical pseudo-stratified epithelium, similar to other mammalian species (Wick and Kress, 2002; Banks, 1992; Junqueira and Carneiro, 2015). Our findings on the prostate and bulbourethral glands of *Leopardus pardalis* complement the findings of Carneiro et al. (2010) for the oncilla (*Leopardus tigrinus*) and *Puma yaguaroundii* (Rocha et al., 2017). Microscopically, it was possible to observe the internal epithelial layer as well as the prostatic concretion, similar to the findings of Wick and Kress (2002) and Junqueira and Carneiro (2015).

The penis was divided into three regions: tail, body and glans and the presence of small spicules was confirmed (Queiroz, 2003; Rocha et al., 2017). Authors relate the function of these spicules to stimulation of the female reproductive tract to accelerate copulation and enhance peristalsis to move sperm through the female reproductive tract (Harcourt and Gardiner, 1994). In cross-section it can be observed that the cavernous bodies are surrounded by tunica albuginea, erectile tissue, deep artery, complementing the findings of Carneiro et al. (2010) for this species.

### Female reproductive system of the ocelot

The shape and color of the ovaries was as described generically to the others mammals by (Derivaux, 1980), Slatter (2007) and Fossum (2008) in domestic felines; Dyce et al. (2010) in domestic felines and canines and crab-eating fox (*Cerdocyon thous*) by Machado et al. (2017). Microscopic analysis of the ovary revealed a medullary region composed of loose connective tissue, a stroma rich in blood vessels and a parenchyma surrounded by a tunica albuginea, structures described in domestic cats and dogs by Dyce et al. (2010), Junqueira and Carneiro (2015) and crab-eating fox by Machado et al. (2017). The oviducts are small with a pink coloration, conformation and anatomical positioning similar to the findings of Dyce et al. (2010) in domestic canids and felines and Machado et al. (2017) in crab-eating fox. The importance of this organ and continuous studies must be highlighted (Andrade et al., 2019).

The uterus *of Leopardus pardalis* is bicornuate as previously described by Slatter (2007) and Fossum (2008) and Dyce et al. (2010) in domestic felines. It has characteristics similar to the uterus of the crab-eating fox (Machado et al., 2017) as well as to the uterus of domestic felines and canids (Atwood, 1955; Schwarze and Schroder, 1970; Nickel et al., 1979; Getty, 1987; Konig and Liebich, 2004). The uterine body is small also in agreement with Slatter (2007) and Fossum (2008) in domestic felines; Dyce et al. (2010) in domestic felines and canids and similar in crab-eating fox (Machado et al., 2017). Miscroscopically, it is possible to observe the presence of a smooth muscle, also called the tunica mucosa or myometrium, which is the innermost muscular layer of the uterus. The presence of uterine glands indicates that the tissue analyzed does not comprise the region of the uterine body, because it is a region absent from glands, previously known from domestic canids and felines (Schwarze and Schroder, 1970; Nickel et al., 1979; Derivaux, 1980; Banks, 1992; Gartner and Hiatt, 1999; Konig and Liebich, 2004; Slatter, 2007; Fossum, 2008; Dyce et al., 2010; Junqueira and Carneiro, 2015) and the crab-eating fox (Machado et al., 2017). Macroscopically, the cervix corresponds to the uterine entry, presenting a musculature thicker than that presented by the uterine body and the vagina, presenting at the junction vaginal uterus, a knotlike aspect, according to the descriptions performed by Slatter (2007) and Fossum (2008) in domestic felines. The internal orifice of the cervix is positioned dorsally and the internal orifice ventrally to the floor of the vagina, in the same way as described by Slatter (2007), Fossum (2008) and Dyce et al. (2010) in domestic felines. The vagina extends to the external urethral ostium and has a thick muscular wall. The vulva comprises vulvar lips and a prominent clitoris. These observations are in agreement with Slatter (2007) and Fossum (2008) in domestic felines, Dyce et al. (2010) in domestic felines and canids and Machado et al. (2017) in the crab-eating fox.

Although there are studies on the macroscopic description of the male reproductive system of *Leopardus pardalis*, the present study sought to complement previous findings based on the microscopic description by histological technique (HE). Regarding the morphology of the female reproductive system of *Leopardus pardalis*,it is important to note that there are studies that have not been published so far, which demonstrates the importance of the work presented here.

The key points and specific findings of this study demonstrated not only the importance of the morphological description of this species, based on a new macroscopic study, but also aimed at the ultrastructural description of the reproductive system of males and females of *Leopardus pardalis*. It is important to note that the microscopic description by classical histological technique (Hematoxylin and Eosin), performed in this study is unprecedented, of great importance and will serve as a complement to the research carried out up to this moment.

## Conclusion

This research emphasizes the importance of the macro- and microscopic description of the male and female reproductive system of *Leopardus pardalis*. The information obtained from the present study made it possible to fill in the gaps that often hinder the reproductive management of this species and also to complement previous macroscopic studies that have been carried out on the species in question. From the insertion of microscopic studies accomplished by classical techniques of histology, it was possible to amplify the knowledge, ultra-structurally. However, we emphasize the importance of further studies on the species in question (scanning electron microscopy, transmission electron microscopy, for example), with contributions to the maintenance of biodiversity and preservation of this species.
